# Tritium-Labeled Compounds III. Aldoses–*1-t*^1^,^2^

**DOI:** 10.6028/jres.064A.018

**Published:** 1960-04-01

**Authors:** H. S. Isbell, H. L. Frush, N. B. Holt, J. D. Moyer

## Abstract

In a new method for preparing aldoses labeled with tritium at carbon 1, the aldonic lactone is reduced with lithium borohydride-*t* in pyridine solution. The method is suitable for preparing aldoses-*1-t* having high specific activities. The crude product contains a substantial proportion of the corresponding alditol-*1-t*, but the pure aldose-*1-t* is readily separated by fractional recrystallization or paper chromotography. By means of an isotope-dilution technique, yields were determined for the following aldoses-*1-t* and the corresponding alditols-*1-t*: d-arabinose-*1-t*, d-xylose-*1-t*, d-ribose-*1-t*, d-glucose-*1-t*, d-galactose-*1-t*, d-mannose-*1-t*, l-rhamnose-*1-t*, maltose-*1-t*, and lactose-*1-t*.

These labeled materials were also prepared by reducing the corresponding lactones with sodium amalgam in tritiated water. Although this latter method is not suitable for preparing labeled aldoses of high specific activity, the products are more readily purified than those obtained by reducing the lactones with lithium borohydride-*t*.

d-Glucose-*1-t*, obtained by each of these reduction methods, was oxidized with bromine, and the resulting d-gluconic acid was found to be nonradioactive. Hence, in the samples oxidized, tritium was present only at C1.

An apparatus used tor reclaiming tritiated water by freeze-drying is depicted; it incorporates an efficient device for trapping entrained solids or liquids.

## 1. Introduction and Discussion

Tritium-labeled organic compounds offer vast opportunity for research in chemistry, biology, and medicine; they can be used not only to follow the course of a chemical or biological process, but also to study the behavior of hydrogen in the process. Extremely important differences in the rates of reaction for tritium and hydrogen provide a means for studying the mechanisms of chemical reactions. Because of the potential usefulness of carbohydrates posit ion-labeled with tritium, the Organic Chemistry Section of the National Bureau of Standards has undertaken a broad program on the development of methods for their preparation and use [1, 2].^3^ Previous publications have reported a simple procedure for the analysis of nonvolatile, water-soluble tritium compounds in a gas-flow, windowless, proportional counter [3], and a general-purpose manifold for conducting reactions in a closed system; in addition, detailed directions have been given for preparing lithium borohydride-*t* [4]. The present report describes methods for preparing aldoses labeled with tritium at C1.

In order to prepare aldoses-*1-t* of high activity, a process was developed in which the aldonic lactone is reduced with lithium borohydride-*t*. Previously, Wolfrom, and coworkers [5, 6] had prepared nonradioactive aldoses by reducing aldonic lactones with nonradioactive sodium borohydride. In a (presumably) similar process, Friedberg and Kaplan [7] had obtained d-glucose-*1-t* by reducing d-glucono-*δ*-lactone with sodium borohydride-*t* Apparently, *lithium* borohydride had not been employed for reducing aldonic lactones to aldoses. The ease of preparation of tritiated lithium borohydride made its use for this purpose attractive. A study of the reduction of d-glucono-*δ*-lactone with lithium borohydride, in various solvents, showed that a fair yield of the aldose could be obtained by the dropwise addition of a pyridine solution of lithium borohydride to the lactone freshly dissolved in ice water. Pyridine appears to alter the reducing properties of the borohydride so as to decrease the tendency of the reagent to reduce the aldose to the alditol. Nevertheless, the crude product contains more or less of the alditol, depending upon the particular lactone reduced and the proportion of borohydride used. Pyridine solutions of lithium borohydride have the additional advantage of being reasonably stable and easy to handle.

Aldoses-*1-t* of low activity were prepared by reducing aldonic lactones with sodium amalgam,^4^ essentially by the method developed in this laboratory for preparing C^14^-labeled aldoses [10, 11, 12]; water-*t* was the solvent, and the reaction mixture included an acid buffer (sodium binoxalate). After the reduction step, the reaction mixture was freeze-dried, and the water-*t* recovered from one preparation was used in the next. Slight dilution of the tritium arose from hydrogen-tritium exchange between the water-*t* and the materials dissolved in it. The tritium-labeled aldoses, after removal of labile tritium by the freeze-drying technique, were separated and purified by the procedures used in preparing the corresponding C^14^-labeled aldoses.

The reduction of aldonic lactones with sodium amalgam does not require elaborate equipment, and is highly satisfactory for obtaining low-activity, tritium-labeled sugars (except tetroses); it is unsatisfactory for preparing materials of high activity.

When samples of d-glucose-*1-t*, prepared by each method of reduction, were oxidized with bromine [13], the resulting barium d-gluconate was found to be nonradioactive. Hence, the d-glucose had been labeled exclusively at C1. Presumably, other sugars prepared by the same reduction methods are also labeled with tritium at C1 only.

## 2. Experimental Procedures

### 2.1. Reduction of Aldonic Lactones With Lithium Borohydride-*t* in Pyridine; Determination of the Yields of Aldoses-*1-t* and of Alditols-*1-t* by the Isotope-Dilution Technique

Under an efficient hood, the reductions were conducted in a 50-ml, round-bottomed flask equipped with a magnetic stirrer and cooled in an ice bath. One millimole of lactone was efficiently stirred in the flask with 1 ml of ice water; immediately thereafter, 1 ml of a dry pyridine solution containing 0.25 millimole of lithium borohydride-*t* (having 160 *μ*c of radioactivity) was added by drops. The mixture was kept in an ice bath for 2 hr and was then allowed to warm to room temperature. The water and pyridine were evaporated in a stream of air, and labile tritium was removed from the residue by dissolving it in 5 ml of methanol and evaporating the solvent; the latter process was repeated four times. Filially, the residue was dissolved in sufficient water to yield a total volume of 10 ml.

A 0.01-ml sample was used for studying the products by paper chromatography; the remainder, divided into two equal portions, was used for determining aldose-*1-t* and alditol-*1-t* by isotope dilution. To one portion was added 200 mg of the nonradioactive aldose, and to the other, 200 mg of the nonradioactive alditol. Each solution was deionized by passing it through a column containing 5 ml of mixed anion- and cation-exchange resins, and the effluent was concentrated substantially to dryness in a rotary still. After three successive recrystallizations of the residue from methanol by the addition of 2-propanol, the diluted aldose or alditol was assayed for radioactivity in a carboxymethylcellulose film [3]. The amount of the radioactive constituent in the diluted product was calculated from the relationship:

Ax=B(x+200)

where *x* is the weight of the radioactive constituent in the aliquot of the original reduction product, *A* is the specific activity of this constituent, and *B* is the specific activity of the diluted constituent. Quantity *A* is calculated on the assumptions (a) that the hydrogen-*t* of the reduction product has the same activity as the hydrogen-*t* of the lithium borohydride- *t* (i.e., that there is no isotope effect in the reduction) and (b) that the aldose contains one atom of hydrogen-*t* and the alditol, two atoms. The yields of aldoses-*1-t* and alditols-*1-t* formed by the reduction of a series of aldonic lactones with lithium borohydride-*t* in pyridine are given in table 1.

### 2.2 Large-Scale Preparation of Aldoses-*1-t* by Reduction of Aldonic Lactones With Lithium Borohydride-*t*

By essentially the same procedure as that described in section 2.1, but by use of a more highly radioactive lithium borohydride-*t* in a closed system [4], 0.5-g quantities of the tritium-labeled aldoses listed in table 1 were prepared; the specific activities were approximately 20 *μ*c/mg.

**Figure 1:**
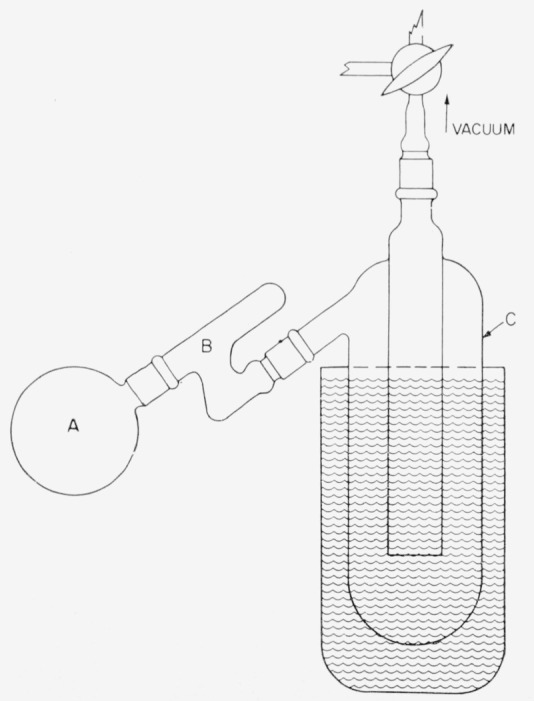
Freeze-drying apparatus with trap for reducing mechanical losses.

Four millimoles of the aldonic lactone and a small stirring magnet were placed in a 100-ml, standard-taper flask having a side arm for the introduction of reagents through a rubber diaphragm (fig. 4 of ref. 4). The flask was attached to the general-purpose manifold of the apparatus of figure 1, reference 4, and evacuated. The connection to the manifold was closed, the flask was immersed in an ice bath set on a magnetic stirrer, and 2 ml of ice water was injected into the side-arm through the rubber diaphragm. While the lactone solution was efficiently stirred, 2 ml of a pyridine solution containing 1 millimole of lithium borohydride-*t* was injected into the system from a microburet. The mixture was kept at 0° C for 1 hr and at room temperature for 5 hr; it was then treated with approximately 1 ml of 10-percent aqueous acetic acid. The flask was immersed in liquid nitrogen, and the hydrogen-*t*, generated in the reaction was transferred by means of the Toepler pump to a flask attached to the manifold; finally, the reaction flask was removed from the manifold. The solution was passed through 10 ml of cation-exchange resin, and then both labile tritium and boric acid were removed by several successive evaporations of methanol solutions to near-dryness. The residue, together with 500 mg of the nonradioactive aldose, was dissolved in water, and the solution was deionized with 10 ml of mixed anion- and cationexchange resins. The salt-free solution,^5^ on concentration and addition of such solvents as methanol, ethanol, or 2-propanol, gave the crystalline aldose-*1-t*, in some cases contaminated with the corresponding alditol-*1-t*. Each product was recrystallized three times from methanol, usually with the addition of 2-propanol.

In a few preparations, difficulty was encountered in separating the aldose from the alditol. The amount of alditol-*1-t* remaining in the aldose-*1-t* after three recrystallizations was determined by the isotope-dilution technique. In each case, 2 mg of the aldose-*1-t* was mixed with 100 mg of the corresponding, nonradioactive alditol. Radioassay of the carrier alditol [3] after three recrystallizations showed that the following aldoses were pure: l-arabinose-*1-t*, d-xylose-*1-t*, d-glucose-*1-t*, l-rhamnose-*1-t*, maltose- *1-t*, and lactose-*1-t*. However, the alditol carriers derived from d-ribose-*1-t*, d-galactose-*1-t*, and d-man- nose-*1-t* showed a radioactivity indicating the presence of substantial quantities of the corresponding alditol-*1-t*. These sugars were then purified by large-scale paper chromatography. The separations will be described in a later publication.

### 2.3. Preparation of Aldoses-*1-t* by Reduction of Aldonic Lactones With Sodium Amalgam in Tritiated Water

The reductions were first performed in a tube of the type developed for use in the synthesis of C^14^- labeled sugars [11, 12].^6^ To the tube, cooled in an ice bath, were added 4 millimoles (712 mg) of d-glucono-*δ*-lactone and 4 g of sodium binoxalate. Twenty milliliters of water-*t* (having 2.2 me of radioactivity per ml) was added through the side- arm, and then while the mixture was vigorously stirred, 9.2 g of 5-pcrcent sodium amalgam in the form of pellets [14]. After all of the amalgam had reacted (about 1 hr), the reaction mixture was transferred to a 250-ml, round-bottomed flask (flask A of fig. 1) 7 and freeze-dried. The water-*t* that collected in trap C of figure 1 was used for a subsequent preparation. The freeze-dried products from 10 successive, similar reductions were combined and neutralized with aqueous sodium hydroxide. By repeated concentration of the solution, and addition of methanol, three crops of salts (sodium oxalate and sodium gluconate) were precipitated. Finally, the mother liquor was de-ionized by means of a column of mixed ion-exchange resins. The solution, when free from ionic impurities, was concentrated to a sirup, from which *α*- d-glucose-*1-t* was crystallized by the addition of methanol, and then of 2-propanol. The recrystallized aldose-*1-t* from the combined reduction products weighed 4.99 g (corresponding to a 68.5-percent yield) and had an activity of 0.08 *μ*c/mg [3]. By use of nonradioactive d- giucose as carrier, additional *α*- d-glucose-*1-t*, equivalent in activity to 0.75 g of the first crop, was obtained. Thus, the yield of *α*- d-glucose-*1-t* was 80 percent of the lactone used.

All of the lactones 8 listed in table 1 were reduced with sodium amalgam by a procedure similar to that described above, but on a larger scale. The preparations were conducted in a 500-ml, two-necked, round-bottomed, stainless-steel flask, equipped with an efficient mechanical stirrer and cooled in an ice bath. One hundred milliliters of water-*t* and 25 to 50 millimoles of lactone were used in each reduction. For all of the aldoses except d-ribose, yields of 50 to 75 percent were obtained, without carrier. The yield of d-ribose was about 35 percent.

### 2.4. Proof of the Position of Label in d-Glucose-*t*; Oxidation With Bromine

A mixture of 45 mg of d-glucose-*t*, 150 mg of barium benzoate, and 3 ml of water saturated with bromine was sealed in a glass tube and kept in the dark, at room temperature, for 18 hr. The contents of the tube were mixed with about 100 mg of a decolorizing carbon and then with a solution containing 100 mg of silver sulfate in 10 ml of hot water. The mixture was filtered, and the filtrate was passed through 3 ml of cation-exchange resin. The resulting solution was then extracted with chloroform (to remove benzoic acid), neutralized with barium hydroxide, filtered, and concentrated. The barium d-gluconate was crystallized and recrystallized from water by the addition of methanol. The product showed no radioactivity when assayed in a film of sodium *O*-(carboxymethyl)cellulose.

## Figures and Tables

**Table 1:** Yields of aldoses-1-t and alditols-1-t obtained by reducing aldonic lactones with lithium borohydride-t^a^

Lactone	Aldose-*1-t*							Alditol-*1-t*, original product	
	
		Diluted product				Original			
		
		Sample counted (*m*′)	cps^b^	Weight film (*m*)	*μ*c/mg^c^ of sample	Yield from 0.5 m. mole lactone^d^	Yield		Yield^e^
									
		*mg*		*mg*		*mg*	%		%
d-Arabono-*γ*-	*β*-d-Arabinosc	0.922	61.7	20.6	0.0613	13.0	17.3	d-Arabitol	8.3
d-Xylono-*γ*-	*α*-d-Xylose	1.146	145.5	20.8	.1180	26.8	35.7	d-Xylitol^f^	4.2
d-Ribono-*γ*-	d-Ribose	0.794	28.7	19.6	.0315	6.5	8.7	d-Ribitol^f^	3.7
d-Glucono-*δ*-	*α*- d-Glucose	1.312	224.5	21.0	.160	47.5	52.8	d-Glucitol	0.6
d-Galactono-*γ*-	*α*- d-Galactose	0.958	92.3	19.8	.0849	22.7	25.2	d-Galactitol^f^	7.6
d-Mannono-*γ*-	*α*- d-Mannose	.838	63.2	20.5	.0688	17.7	19.7	d-Mannitol	1.6
l-Rhamnono-*γ*-	*α*-l-Rhamnose (hydrate)	1.058	87.4	19.9	.0732	19.5	21.4	l-Rhamnitol	1.3
Maltobiono-*δ*-	*β*-Maltose (hydrate)	1.012	136.9	19.8	.119	79.9	44. 4	Maltitol	………
Lactobiono-*δ*-	*α*-Laetose (hydrate)	1.236	174.3	20.9	.131	91.7	50.9	Lactitol (hydrate)	0.2

a One millimole of aldonic lactone was reduced with 0.25 millimole of lithium borohydride-*t* having an activity of 150 *μ*c per milliatom of hydrogen-*t*. The product was divided into two equal portions; 200 mg of carrier aldose was added to one portion, and 200 mg of carrier alditol to the other.

b The sample, in a film of sodium *O*-(carboxymethyl)cellulose on a planchet, was counted in a windowless, gas-flow, proportional counter.

c Calculated from the equation $\mu c/\text{mg}=\frac{\text{cps}\times m\times k}{m^{\prime }}$, where cps is the observed counts per second, and *k* is an empirically determined constant, in this case, 4.45×10^−5^.

d Calculated from the equation above. Quantity A, in *μ*c/mg, is based on the activity of the lithium borohydride-*t*. (See footnote a). Quantity B, in *μ*c/mg, is the activity of the diluted product. (See previous column.)

e Yield of alditol-1-*t* was determined by the method shown in detail for the aldose-1-*t*, but with allowance for the fact that *two* atoms of hydrogen-*t* were introduced into the molecule of the alditol.

f Because of the tritium label at C1, the compound is not *meso.we*

## Footnotes

4 Topper and Stetten [8] had previously prepared d-glucose-*1-d* by reducing d-glucono-*δ*-lactone with sodium amalgam in deuterated water. They used Fischer’s method of reduction [9], in which the acidity is maintained by the drop- wise addition of mineral acid.

5 Before concentration, solutions were tested for ionic impurities with a conductivity meter.

6 The tube, which was 20 cm long and 2.5 cm wide, had an oblique side-arm near the top for the addition of amalgam; the tube was equipped with a stainless- steel stopper, through which a stainless-steel stirrer extended to the bottom.

7 The freeze-drying apparatus of figure 1 depicts a highly efficient trap B, in which entrained material is caught in the side-arm. It may then be recovered by dissolving it in a solvent, and removing it with a capillary pipet.

8 L-Arabono-*γ*-lactone was reduced, instead of the d form listed in the table.
